# Inverse association between regional solar radiation and hospital discharges due to bullous diseases in Chile: An ecological study

**DOI:** 10.1016/j.jdin.2024.09.003

**Published:** 2024-10-10

**Authors:** Ignacio Alarcón, Javier Arellano

**Affiliations:** a Escuela de Medicina, Facultad de Medicina, Universidad de Chile, Santiago, Chile; b Jefe de Dermatología/UNACESS, Hospital Clínico San Borja - Arriarán, Santiago, Chile; c Servicio de Dermatología, Facultad de Medicina, Universidad de Chile, Santiago, Chile

**Keywords:** bullous diseases, ecological study, pemphigoid, pemphigus, solar radiation, vitamin D

*To the Editor:* Over the last years, multiple studies have sought to find associations between vitamin D levels and various diseases (eg, inflammatory and autoimmune diseases). Countless functions of vitamin D in immunity, different from the well-known osteometabolic functions, have been described.^1^^,^^2^ For this reason, there has been interest in evaluating the possible association between vitamin D and bullous diseases.

Bullous diseases have a wide range of manifestations and can be categorized according to the level of the skin where the blister occurs, either intraepidermal (pemphigus) or subepidermal (pemphigoid).^3^ Unlike its clinical and histopathological aspects, there is limited amount of literature regarding their epidemiological characteristics, and studies are generally small with a high risk of bias.

To date, studies have evaluated the possible association between individual vitamin D levels and the incidence of bullous diseases, with heterogeneous results.^4^ Moreover, studies have included patients from the same geographical location, lacking sufficient heterogeneity of exposure.

In this context, Chile's unique geography, located between latitudes 17° S and 56° S, is highly advantageous as it allows the population to have different levels of solar radiation exposure across various latitudes and regions. This, in turn, suggests different levels of vitamin D in the population, achieving a broad range of exposure heterogeneity. Previous works explore the association between dermatological pathologies and lower levels of vitamin D in Chile, correlated with latitude and exposure to UV radiation.^5^

We performed an ecological study to evaluate the association between regional solar radiation and hospital discharges due to bullous diseases in Chile between 2001 and 2015. Hospital discharge rates for bullous diseases were calculated per million inhabitants and analyzed concerning annual cumulative solar radiation, finding a statistically significant inverse relationship between these 2 variables.

Regions with higher levels of solar radiation had lower rates of hospital discharges for these conditions. This trend was consistent across different age groups, with a particularly notable effect in the population aged 50 years and older. The regression analysis showed a negative coefficient for annual cumulative solar radiation, indicating that as solar radiation increases, the rate of hospital discharges decreases (*P* < .05) (Fig 1). This can be observed in colorimetric maps that visually represent the regional variations in these figures (Fig 2).

**Fig 1:**
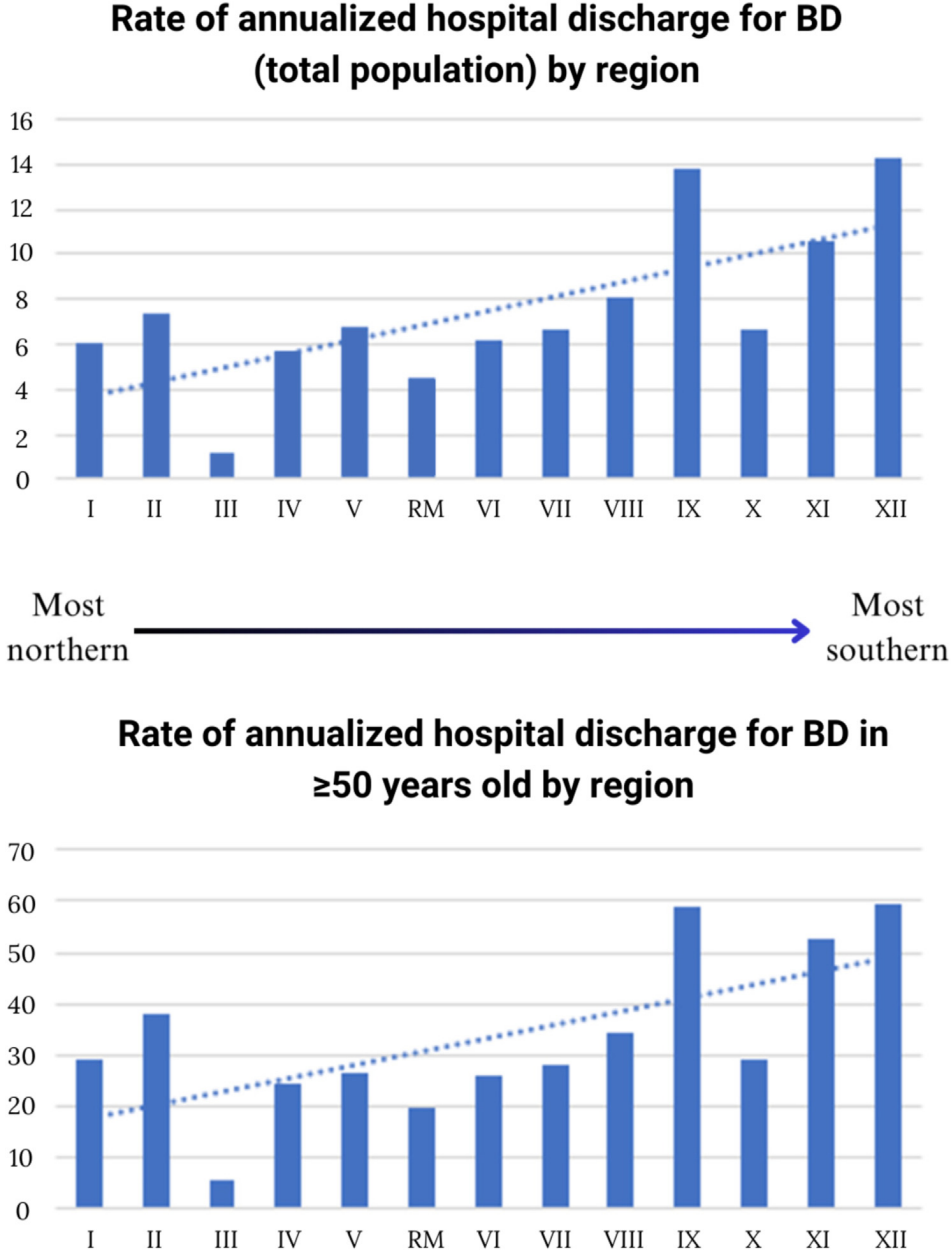
Graphs showing discharge rates for bullous diseases by region from 2001 to 2015 (rates per 1 × 106 inhabitants), for the total population and those over 50 years of age, demonstrating a linear association trend between higher rates and higher latitudes.

**Fig 2:**
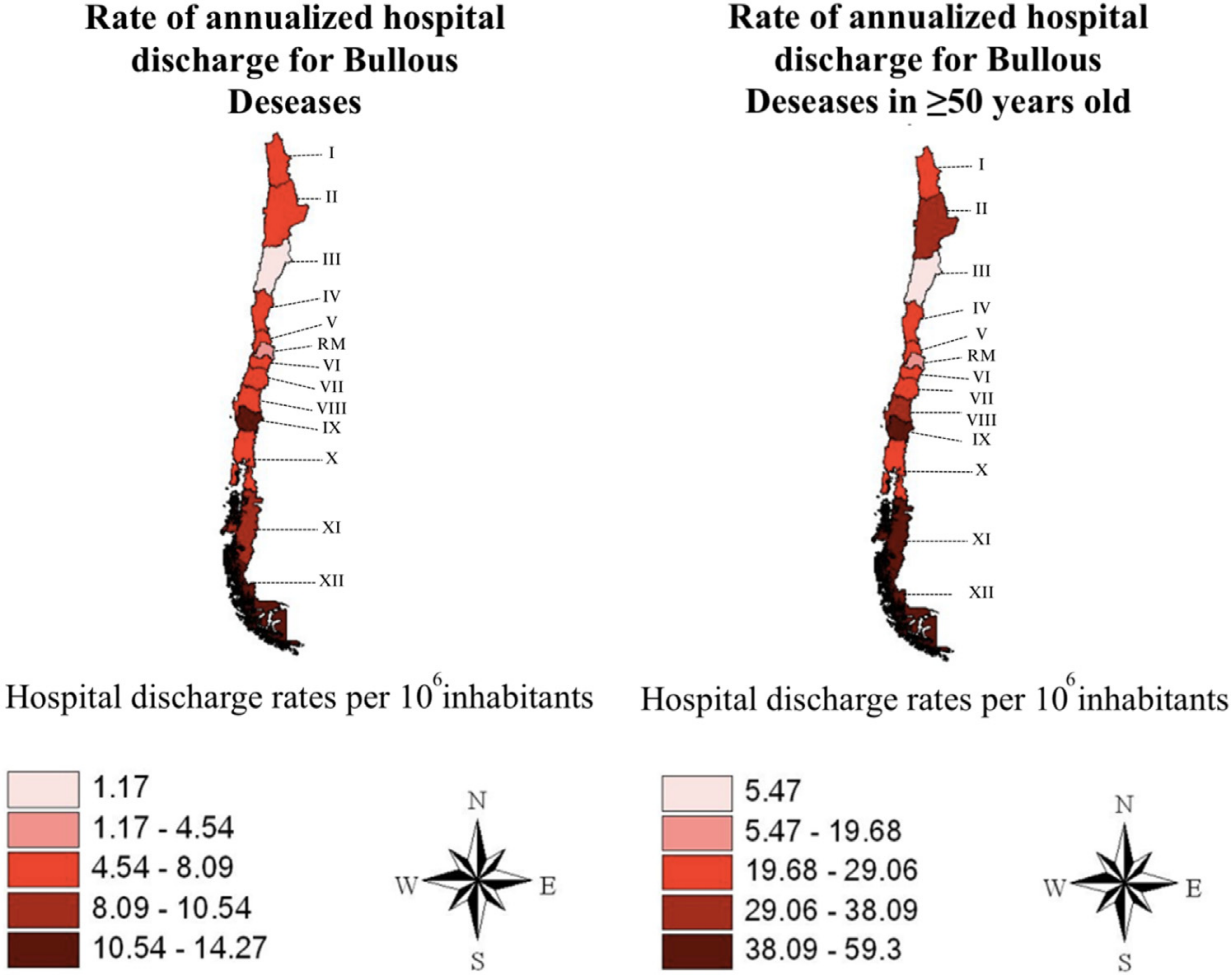
Colorimetric maps of hospital discharge rates for bullous diseases in the total population and in those over 50 years of age showing a gradient indicating a trend toward higher rates at higher latitudes.

These findings suggest that higher solar radiation may have a protective effect against the development or exacerbation of bullous diseases. Possible mechanisms include the immunomodulatory effects of ultraviolet radiation. However, further research is needed to elucidate the implicated pathways and confirm it in different populations and contexts.

This study provides preliminary evidence of an inverse association between solar radiation and hospital discharges due to bullous diseases in Chile, which might be related to the immune functions of vitamin D. However, these results should be interpreted with caution due to its ecological-level nature: our results provide an indication of the association that we might find at individual level, which has been demonstrated in some previous studies and could be further complemented by more individual observational studies. This could help us define future intervention strategies for managing patients with bullous diseases.

## Conflicts of interest

None disclosed.
